# Small Improvements in Postoperative Outcome with Gap Balancing Technique Compared with Measured Resection in Total Knee Arthroplasty

**DOI:** 10.2174/1874325001711011236

**Published:** 2017-11-10

**Authors:** Hagen Hommel, Daniel Kunze, Peggy Hommel, Peter Fennema

**Affiliations:** 1 KH-MOL GmBH Sonnenburger Weg 3, 16269 Wriezen, Germany; 2 Medizinische Hochschule Brandenburg (MHB), Theodor Fontane, Fehrbelliner Straße 38, 16816 Neuruppin, Germany; 3 AMR Advanced Medical Research GmbH, Hofenstrasse 89b, 8708 Männedorf, Switzerland

**Keywords:** Osteoarthritis, Knee, Total knee arthroplasty, Ligament balancing, Gap balancing, Measured resection, Randomized clinical trial

## Abstract

**Background:**

There is ongoing debate about how to obtain correct rotational alignment in total knee arthroplasty (TKA). Two commonly used techniques are the measured resection (MR) and the gap balancing (GB) technique.

**Objective:**

The objective of the present study was to analyze which of these two techniques confers a clinical advantage up to 10 years postoperatively.

**Methods:**

Two hundred patients were randomized to either MR or GB. The primary outcome was the Knee Society Knee Score (KS) 10 years postoperatively. Secondary outcomes were passive range of motion, the Knee Society Function Score (FS), and the Western Ontario and McMasters Universities Osteoarthritis Index (WOMAC), along with implant survival. We employed a two one-sided test (TOST) and linear mixed models to assess clinical outcomes.

**Results:**

Mean KS was 82 (95% confidence interval (CI), 80 – 83) and 77 (95% CI, 76 – 79) in the GB and MR group, respectively. The TOST test and linear mixed model both revealed statistical significance (p < 0.001). In addition, GB yielded better postoperative FS and WOMAC. However, between-group differences were consistently small. Implant survival rates at 10 years, with survival for any reason as the endpoint of interest, were 93.7% (95% CI, 86.4% and 97.1%) and 89.8% (95% CI, 81.9% - 94.4%) for the GB group and the MR group, respectively (*p* = 0.302).

**Conclusion:**

Gap-balancing is a safe and reliable technique. KS for the two study groups at 10 years can be considered equivalent, and the small postoperative advantages may not extend beyond clinical relevance.

## 1 INTRODUCTION

The incidence of gonarthrosis and the subsequent demand for total knee arthroplasty (TKA) is increasing due to the aging population and the exponential increase in obesity [1]. The revision burden after TKA is approximately 8%, with complications such as instability, malalignment, and malrotation being contributing factors [1, 2]. Additionally, approximately 20% of TKA patients are dissatisfied with the procedure due to residual pain, limited function, and occasional instability [3]. Malrotation and malalignment are thought to contribute to this rate [4, 5], underlining the importance of optimal implant alignment and rotation [6, 7]. In order to correct the deformity, equalize the flexion and extension gaps, and restore stability, it is essential that there is accurate resection of bone and soft-tissue balancing [8]. Correct alignment and balance between the flexion and extension gaps are necessary before there can be a satisfactory outcome. Computer navigation has improved coronal and sagittal alignment [9, 10], but this technique does not improve rotational alignment [10]. Rotational alignment of the femoral component is determined by anterior and posterior femoral condylar osteotomy. A rotational malalignment can lead to patellofemoral complications, such as limited movement, anterior knee pain, subluxation, maltracking, and loosening. Gap balancing (GB) and measured resection (MR) are two techniques that are widely used to confirm rotational alignment [11]. GB involves release of soft tissue in order to achieve balance in flexion and extension followed by equalization of the gap for determining the resection of bone [11]. MR uses anatomical landmarks, such as the posterior femoral condylar axis and/or the transepicondylar and anteroposterior axes, to direct the resection of bone followed by soft-tissue release to ensure equalization of the extension and flexion gaps [11]. There is controversy over which of these techniques results in the most accurate rotational alignment.

We performed a randomized controlled trial comparing the postoperative outcomes of the MR and GB techniques up to 10 years after surgery. Clinical and functional outcome scores, including the Knee Society Score (KSS), knee flexion, patient satisfaction, and adverse events, were evaluated. We hypothesize that patients operated with the GB technique would achieve a similar KSS Knee Score (KS) as patients operated with a conventional MR technique.

## 2 MATERIALS AND METHODS

**Fig. (1):**
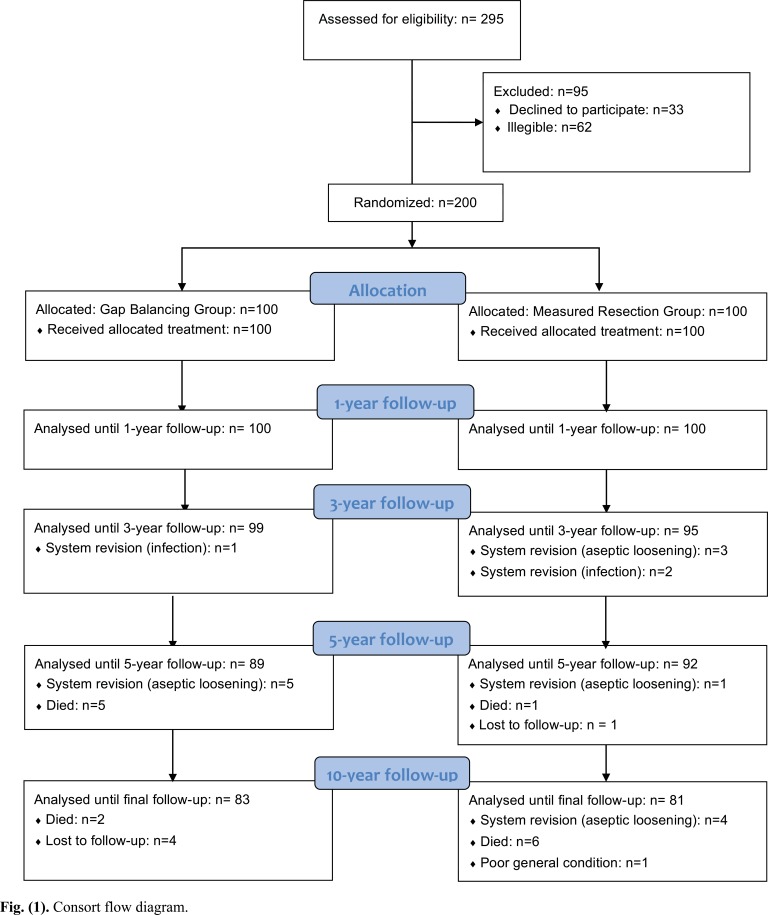
Consort flow diagram.

This is a single-center, prospective, parallel group randomized controlled trial. Equal randomization (1:1) was used (Fig. **1**). Patients, who were recruited from December 2003 to May 2006, had their procedures completed from January 2004 to June 2006. The study protocol was approved by the regional ethics committee. Written informed consent was obtained from all participating patients prior to study commencement.

Adult patients on the waiting list for a unilateral TKA were eligible. Inclusion criteria were as follows: non-inflammatory osteoarthritis, moderate-to-severe pain in the affected knee, absence of large deformities (knee flexion < 90°, fixed flexion deformity > 20°, varus or valgus deformity > 10° [unless correctable < 10°]), a stable knee (no grade-3 collateral ligament insufficiency), and sufficient tibial or femoral bone stock. Exclusion criteria were as follows: rheumatoid arthritis, body mass index > 40 kg/m^2^, hip or knee replacement in the previous 6 months, infection (active, local, or systemic) or an immunosuppressive disorder, and a known sensitivity to the implant biomaterials. In order to get a reasonably homogeneous study population in terms of patient activity, quality of the host bone, and probability of attrition, patients older than 70 years were not eligible for the study. The same postoperative rehabilitation program was used for all patients, which consisted of partial weight bearing with crutches on the first day after surgery and active ROM exercises.

A single surgeon (HH) carried out all of the procedures with general endotracheal anesthesia, and a combined femoral nerve block and sciatic nerve block. A tourniquet was used in every case. A medial parapatellar approach was used for all exposures. All TKAs were peformed using a navigation system (PI Galileo, Smith & Nephew Orthopaedics AG, Baar, Switzerland). Osteophytes were debrided and the patella was everted. In the MR group, conventional measured resection surgical techniques were used, and soft-tissue tension was evaluated and optimized with a block spacer. Femoral component rotation was determined using the posterior condyles as reference. In the GB group, standard instrumentation was used for the proximal tibial and the distal femoral cuts. The “Extension Gap-First technique” was used to perform gap balancing with a balancer device [12]. The balancer device was used to distract the femur from the proximal tibia. Following each soft-tissue release step, the device was used to measure the extension gap until a rectangular extension gap was obtained. A gradual soft-tissue release was performed to obtain a symmetrical extension gap where appropriate [13, 14]. The values established for the extension gap were then applied to the flexion gap. A rectangular flexion gap was achieved by femoral rotation based on the tension of the soft-tissues. Final bone cuts were carried out and implant placement took place as usual. Femoral component rotation was determined intraoperatively by the navigation system. A TC-Plus Solution (Smith & Nephew Orthopaedics AG) cruciate-retaining TKA was implanted in all cases, as this device was routinely used in our clinic at that time point. The tibial components were fixed with cement, whereas the femoral components were implanted cementless. The patella was not resurfaced in any of the cases. The same standardized postoperative multimodal pain protocol was applied for all patients. This consisted of a continuous femoral-sciatic nerve block for the first 24 hours, with subsequent intermittent femoral and sciatic injection on demand. In addition, 4 doses of acetaminophen (1 g) and 2 doses of indomethacin (50 mg) were provided on the first postoperative day. Morphine (for the first 48 hours) or tramadol (after 48 hours) was used for the treatment of pain exacerbations.

The primary outcome was the KS 10 years postoperatively. Secondary outcomes were passive range of motion, the KSS Function Score (FS) [15], the Western Ontario and McMasters Universities Osteoarthritis Index (WOMAC) [16], patient satisfaction, adverse events, and radiographic outcome. Sociodemographic variables and disease-related data were recorded during the baseline assessment. Peri-operative data were collected on duration of surgery (starting from the first incision), approach, blood loss, soft-tissue release, and complications. The independent research physician, who was blinded for the assigned treatment, collected postoperative data at 1, 3, 5, and 10 years after surgery.

The Knee Society Radiographic Evaluation and Scoring System was used to evaluate radiographic parameters [15]. Standardized standing anteroposterior (AP), skyline patella, and lateral radiographs were taken and then measured for radiolucent lines (RLL), focal osteolysis, and evidence of component subsidence. Our definition of focal osteolysis was any progressive osteolytic lesion at the bone–implant interface [17]. Our definition of loss of fixation was a continuous RLL greater than 1 mm in all zones, or a change in the position of the implant [18]. Mechanical axis alignment was measured via long-leg standing lower extremity radiographs both preoperatively and postoperatively. Malalignment was defined as a deviation of greater than 3° from neutral alignment.

The null hypothesis of the study was that GB is nonequivalent to the standard treatment. A difference of 7.5 points in Knee Score was considered clinically important. A sample size of 69 gives a two-group 0.05 one-sided t-test the power to reject the null hypothesis. In order to compensate for an anticipated attrition of 35%, it was intended that an initial sample size of 100 patients would be enrolled in each group.

Categorical variables are presented as frequencies and percentages. Continuous data are presented as mean ± standard deviation (SD) for univariate data or as mean (95% confidence interval) for model-based data. Bivariate analysis was performed using the Chi-squared test or Fisher exact test for categorical variables, and the Student’s t-test for continuous variables.

The primary hypothesis was tested using the two one-sided test (TOST) procedure to test equivalence [19]. In the TOST procedure an upper (δ) and lower (-δ) equivalence bound is specified based on a minimally clinically relevant difference. Two composite null hypotheses are tested: H_o1_: δ - [mean(KS_GB_) - mean(KS_MR_)] ≤ 0; and H_o2_: [mean(KS_GB_) - mean(KS_MR_)] + δ ≤ 0. When both hypotheses are refuted, it is concluded that the observed effect falls within the equivalence bounds and is statistically smaller than the minimally clinically relevant difference, and the interventions can therefore be considered practically equivalent.

We also employed linear mixed regression models to analyse both primary and secondary clinical outcomes over time [20]. Implant survivorship was calculated using Kaplan-Meier analysis [21], with the following events of interest: implant revision due to any reason, and implant revision due to aseptic loosening. Patients who were lost to follow-up were censored from the time point their status was unknown. Logrank tests were used to determine the presence of an association between surgical technique and implant survival.

Stata 12.1 (StataCorp, College Station, TX) was used for the analysis. All analyses were performed in accordance with the intention-to-treat principle, in which the groups were analyzed exactly as randomized. Incidentally missing data were not imputed.

The surgeon could not be blinded for the assigned treatment, but participants were blinded up to the 10-year follow-up assessment. To further avoid bias, the clinical data was collected by an independent blinded research nurse, the radiographic measurements were carried out by an orthopaedic physician assistant who was not involved in the healthcare process, and the statistical analyses were carried out by an independent researcher who was similarly uninvolved in the patient treatment.

## 3 RESULTS

Both patient groups were comparable in terms of gender, age, weight, BMI, and most clinical variables, whereas the FS was slightly better in the GB group (Table **1**). Surgical time in the GB group was 74.4 ± 7.8 minutes, versus 68.4 ± 6.5 minutes in the MR group (*p* < 0.001). Femoral rotation was 2° ± 1° (range, -2° – 5°) external rotation in the GB group and 3° in the MR group. In most of the patients in the GB group, removal of the osteophytes was sufficient for balancing and no additional ligament releases were necessary (Table **2**). Ligament releases were more common in the MR group. There were 83 patients in the GB group and 81 patients in the MR group available for the 10-year analysis (Fig. **1**). A total of 5 patients were lost to follow-up, 4 from the GB group and 1 from the MR group. Overall, 14 patients (7 in the GB group and 7 in the MR group) died before the 10-year follow-up, with all deaths unrelated to the knee. One additional patient in the MR group developed severe dementia and was unable to attend further follow-ups. In the GB group, 5 patients were revised for aseptic loosening and 1 patient for septic loosening. In the MR group, 8 patients required revision for aseptic loosening while 2 patients required revision for septic loosening. Postoperative complications not leading to revision were as follows: In the GB group, one postoperative hematoma and one delayed wound healing was observed. In the MR group, three wound healing disturbances were noted.

Table **3** presents the postoperative KS of the MR and the GB groups. The TOST test was highly significant, indicating that the between-group differences were smaller than the pre-specified margin of indifference. However, the linear mixed-effects regression models revealed a significantly higher KS was found in the GB group at all postoperative time points.

The GB group showed higher postoperative FS and WOMAC scores, and an increased range of motion, but differences were small and did not always reach the level of statistical significance (Table **4**).

Preoperative mechanical alignment was 5.2° ± 4.5° varus in the GB group and 5.4° ± 4.8° in the MR group. Postoperative values were 1.3° ± 1.6° and 1.9° ± 2.0°, respectively (*p* = 0.024). In the GB and the MR group, there were 23 and 35 knees with more than 3° deviation from neutral alignment, respectively (*p* = 0.061), There was no evidence of component subsidence in any of the cases. No osteolysis involving either the tibia or femur was observed in any of the cases. There were 3 cases with tibial RLL and 1 case with femoral RLL in the MR group - these cases were evaluated as radiographically loose.

With survival for aseptic reasons as the endpoint of interest, survival rates were 94.6% (95% CI, 87.5% and 97.7%) and 91.6% (84.0% - 95.7%) for the GB group and the MR group, respectively (*p* = 0.386). With survival for any reason as the endpoint of interest, survival rates were 93.7% (95% CI, 86.4% and 97.1%) and 89.8% (95% CI, 81.9% - 94.4%) for the GB group and the MR group, respectively (*p* = 0.302).

## 4 DISCUSSION

The most important finding of the present study was that the GB group yielded improvements in all clinical parameters postoperatively compared to the MR group. For the primary endpoint the null hypothesis of non-equivalence could be rejected, indicating equivalence in terms of KS for the two surgical techniques at 10 years. However, the linear mixed regression model revealed a significantly higher KS in the GB group. Statistical equivalence in combination with statistically different results implies that results can be interpreted as “Trivially Different” [22], *i.e*., the difference does not extend beyond clinical relevance. The secondary endpoints showed a similar pattern as the primary endpoint, with small differences that did not reach the level of statistical significance at all time points. Moreover, our study did not reveal statistical evidence that any of the two techniques confers survival advantages.

Daines and Hofmann have postulated that the risk of anterior knee pain and/or postoperative stiffness could be minimized using the GB technique [11, 23]. In addition, Daines and Fehring concluded that the GB technique may lead to more physiological kinematics [11, 24]. Although stiffness and kinematics were not directly assessed, the findings in the present study may confirm these potential benefits of the GB technique.

The results of the present study are in agreement with a recently published meta-analysis that showed a mean difference of 2.5 points for the KS and a mean difference of 5.0 points for the FS in favor of the balancing techniques [25]. However, this meta-analysis was based on a small number of included studies, and follow-up was limited to 2 years postoperatively. Furthermore, a significant difference in joint line of 1.12 mm (95% CI, 0.70 to 1.55 mm) was found, with a higher position for the GB groups. All included studies used a tibia first technique, which was attributed to the prioritization of the symmetry of the flexion and extension gaps [25]. Joint line assessment was not undertaken in this study. However, the study cohort did not contain any evidence of postoperative clinical effects or complications, such as mid-flexion instability, patellofemoral pain syndrome, or contractures due to raising the joint line.

However, a highly accurate proximal tibial cut is required for the GB technique because initial tibial alignment errors will be carried through to all subsequent cuts [26]. There is evidence that secondary rotational malalignment of the femoral component can be caused by malalignment of the tibial component in the coronal plane [27]. We previously presented a study that demonstrated that the use of patient-specific instrumentation (PSI) may be advantageous to achieve optimal component positioning. However, recent meta-analyses of studies have shown that PSI does not improve surgical precision - especially for the tibial component [28, 29].

The postoperative mechanical alignment differed slightly between the two groups, but the difference is not thought to be attributable to the surgical technique. Significant differences in terms of mechanical axis outliers were not observed in this study. All knees were clinically stable on clinical examination. In the GB group, femoral rotation was within the range of -2° to 5° of external rotation in all cases. However, when using the GB technique, rotational adjustment deviations of up to 6° are not an uncommon feature and they are not believed to adversely affect outcome [30, 31].

The 10-year survival rates found in this study are somewhat, although not significantly, less than for many modern implant systems, for which 10-year survival rates well above 90% are typically reported [32]. The implant system used in the present study has been used for many years in our clinic, with an overall 10-year survival rate of around 95%. Notably, this is in line with the survival rate reported in the peer-reviewed literature and in the UK National Joint Registry for the study device [18, 32]. It has to be noted that the 10-year survival rates in the present study do not largely differ from the “Benchmark” of 95%, i.e., the upper limits of the confidence intervals were greater than or approached 95%.

There are limitations to our study. Foremost, the variance in clinical outcome in the study population was smaller than anticipated in the *a priori* power analysis. The smaller the variability in patient response, the easier to demonstrate statistical significance. The small clinical differences, therefore, may lack clinical relevance. This was confirmed by the equivalence testing conducted for the KS that indicated the difference was smaller than the pre-specified equivalence limit difference. Second, the minimally clinically important difference of 7.5 for the KS has been questioned recently and has been estimated to be between 5.3 and 5.9 [33]. Third, a single surgeon in a single institution performed all of the procedures. The surgeon has extensive experience with the GB technique, so the findings are not necessarily generalizable.

## CONCLUSION

This study demonstrates that, compared with standard techniques based solely on bony landmarks, kinematic femoral alignment with gap-balancing is a safe and reliable technique. Overall, favorable results were achieved in both groups after 10 years. The KS for the two study groups at 10 years was statistically equivalent for the two groups. However, GB yielded small postoperative advantages in KS, which may not extend beyond clinical relevance. We attribute these differences to gap-balancing allowing anatomically precise three-dimensional reconstruction. The results for FS, KS, and WOMAC were consistent with this conclusion. Differences were small and did not consistently reach the level of significance. Further large-scale and long-term clinical outcome studies evaluating the effect of GB on clinical outcome are warranted.

## Figures and Tables

**Table 1:** Demographic and preoperative variables.

**-**	**MR group** **n=100**	**GB group** **n=100**
Women (%)	63 (63)	62 (62)
Age at TKA [years]^a^	66.9 (6.0)	67.1 (6.4)
BMI [kg/m^2^] ^a^	30.3 (4.0)	29.8 (3.5)
ASA score (1 / 2 / 3)	5 / 49 / 36	10 / 55 / 35
KS^b^	25 (24 – 26)	26 (25 – 26)
FS ^b^	21 (10 – 22)	23 (22 – 24)
WOMAC ^b^	65 (63 – 66)	65 (63 – 66)
Range of motion ^b^	104 (102 – 106)	103 (102 – 105)
Alignment	5.5 (4.8)	5.2 (4.5)

^a^Presented as mean (standard deviation). ^b^Presented as mean (95% confidence interval).

Abbreviations: MR = measured resection, GB = gap balancing, ASA = American Society of Anesthesiologists, BMI = body mass index, KS = knee score, FS = function score, WOMAC = Western Ontario and McMasters Universities Osteoarthritis Index

**Table 2:** Ligament releases.

**-**	**Extension** **n=100**		**Flexion** **n=100**	
**-**	**ExtensionMR Group**	**ExtensionGB Group**	**ExtensionMR Group**	**ExtensionGB Group**
Osteophyte resection only	71	60	100	64
Superficial medial collateral ligament	21	38	0	34
Posterior capsule	8	2	0	2
*p*-value	0.008		< 0.001	

Abbreviations: MR = measured resection, GB = gap balancing

**Table 3:** Knee Score.

**-**	**MR**	**GB**	***p*-value TOST^†^**	***p*-value linear mixed model**
Year 1	87 (86 – 88)	89 (88 – 90)	< 0.001	0.004
Year 3	87 (86 – 88)	90 (89 – 91)	< 0.001	< 0.001
Year 5	82 (80 – 83)	85 (84 – 87)	< 0.001	< 0.001
Year 10	77 (76 – 78)	82 (80 – 83)	< 0.001	< 0.001

All values are mean (95% confidence interval). Abbreviations: MR = measured resection, GB = gap balancing, TOST = two one-sided t-test

**Table 4:** Other clinical outcomes.

**-**	**FS**		**-**	**WOMAC**		**-**	**ROM [°]**		**-**
**-**	**MR**	**GB**	***p*-value**	**MR**	**GB**	***p*-value**	**MR**	**GB**	***p*-value**
Year 1	86.3 (84.7 – 87.8)	88.6 (87.3 – 90.0)	0.023	30.1 (28.2 – 32.0)	28.5 (26.7 – 30.2)	0.206	103.3 (100.7 – 105.8)	109.5 (107.1 – 111.9)	< 0.001
Year 3	86.3 (84.7 – 87.8)	89.3 (87.9 – 90.7)	0.004	30.6 (28.6 – 32.5)	27.7 (26.0 – 29.5)	0.030	105.4 (102.8 – 108.1)	111.3 (108.8 – 113.7)	< 0.001
Year 5	82.0 (80.3 – 83.8)	83.9 (82.3 – 85.4)	0.125	33.8 (31.8 – 35.8)	30.7 (28.9 – 32.6)	0.023	109.6 (106.7 – 112.5)	110.7 (107 – 112)	0.588
Year 10	77.5 (75.7 – 79.3)	79.9 (78.3 – 81.4)	0.049	36.7 (34.7 – 38.7)	33.7 (31.9 – 35.6)	0.035	111.7 (108.8 – 114.7)	111.4 (108.8 – 114.0)	0.857

All values are mean (95% confidence interval). Abbreviations: ROM = range of motion, MR = measured resection, GB = gap balancing, FS = function score, WOMAC = Western Ontario and McMasters Universities Osteoarthritis Index
